# *N*-Acetyl Cysteine Supplement Minimize Tau Expression and Neuronal Loss in Animal Model of Alzheimer’s Disease

**DOI:** 10.3390/brainsci8100185

**Published:** 2018-10-11

**Authors:** Teresa Joy, Muddanna S. Rao, Sampath Madhyastha

**Affiliations:** 1 Department of Anatomy, Kasturba Medical College, Mangalore 576104, India; teresa.joy@manipal.edu; 2 Manipal Academy of Higher Education, Mangalore 576104, India; 3 Department of Anatomy, Faculty of Medicine, Kuwait University, Kuwait City 13110, Kuwait; muddanna@HSC.EDU.KW

**Keywords:** hippocampus, medial prefrontal cortex, *N*-Acetyl cysteine, neurofibrillary tangles, oxidative stress, tau protein

## Abstract

Alzheimer’s disease (AD) is characterized by the accumulation of neurofibrillary tangles (NFT), deposition of beta amyloid plaques, and consequent neuronal loss in the brain tissue. Oxidative stress to the neurons is often attributed to AD, but its link to NFT and β-amyloid protein (BAP) still remains unclear. In an animal model of AD, we boosted the oxidative defense by *N*-Acetyl cysteine (NAC), a precursor of glutathione, a powerful antioxidant and free radical scavenger, to understand the link between oxidative stress and NFT. In mimicking AD, intracerebroventricular (ICV) colchicine, a microtubule disrupting agent also known to cause oxidative stress was administered to the rats. The animal groups consisted of an age-matched control, sham operated, AD, and NAC treated in AD models of rats. Cognitive function was evaluated in a passive avoidance test; neuronal degeneration was quantified using Nissl staining. NFT in the form of abnormal tau expression in different regions of the brain were evaluated through immunohistochemistry using rabbit anti-tau antibody. ICV has resulted in significant cognitive and neuronal loss in medial prefrontal cortex (MFC) and all the regions of the hippocampus. It has also resulted in increased accumulation of intraneuronal tau in the hippocampus and MFC. NAC treatment in AD model rats has reversed the cognitive loss and neuronal degeneration. The intraneuronal tau expression also minimized with NAC treatment in AD model rats. Thus, our findings suggest that an antioxidant supplement during the progression of AD is likely to prevent neuronal degeneration by minimizing the neurofibrillary degeneration in the form of tau accumulation.

## 1. Introduction

Alzheimer’s disease (AD) is one of the most devastating neurodegenerative disorders, with profound medical and social consequences. It is the most common cause of disability in aged individuals. The two-major pathophysiologies of the disease are the intraneuronal accumulation of hyper phosphorylated tau protein in the form of neurofibrillary tangles (NFT) and abnormal extra cellular deposition of beta amyloid proteins (BAP). These two phenomena are mainly initiated and enhanced by oxidative stress [1]. In aging brain, oxidative stress is because of an increase in oxidant contents and minimized antioxidant defense. The overproduction of reactive oxygen species (ROS) causes neuronal damage and/or death [2].

Colchicine is a microtubule distracting agent, known to cause damage to the neurons. It brings neurofibrillary disintegration by binding to tubulin, the structural protein of the microtubule. Colchicine neurotoxicity in the form of BAP expression [3], free radical production in brain [4,5], loss of cholinergic neurons [6], and memory impairment [7] is known, and it closely simulates human AD [8]. Tau is the major microtubule associated phosphoprotein in neurons; its role in assembling of microtubules depends on its degree of phosphorylation. In AD brain, tau is hyper-phosphorylated, and is polymerized into paired helical strands blended with straight filaments creating NFT. In an earlier study, intrathecal colchicine has induced NFT in motor neurons [9]. It has been claimed that ICV colchicine-induced tau pathology is due to dephosphorylation, rather than hyperphosphorylation [10].

The relationship between oxidative stress and tau neuropathy is not clear. The accumulation of hyper phosphorylated tau is known to cause oxidative stress, but ROS have also been shown to stimulate tau hyperphosphorylation [11]. Increased ROS production and tau hyperphosphorylation were observed in mice hippocampus after treatment of 1,2-diacetylbenzene, a neurotoxic metabolite [12]. Hence, oxidative stress and tau hyperphosphorylation are the two key components of a vicious circle that plays a crucial role in the pathologic process of AD.

AD is characterized by neuronal loss in the hippocampal and temporal lobes, sparing the frontal lobes initially. Medial and orbitofrontal cortices [13] receive direct input from CA1 region of the hippocampus [14]. Because of these connections, the frontal cortex, especially medial and orbital, becomes pathologically involved in dementia associated with AD [15]. According to the connection hypothesis postulated by many researchers, atrophy might also progress to the medial frontal cortex (MFC). 

In continuing this oxidative stress theory of AD, several antioxidant substances have been tested in different models of tauopathies, showing interesting results. Antioxidant therapies like vitamin C, vitamin E [16], and curcumin [17] have also been shown to inhibit the progression of tau pathology. NAC is a derivative of amino acid cysteine, a precursor in the formation of the antioxidant glutathione in the body. NAC is a membrane-permeable cysteine forerunner that does not require active transport. Once it has entered into the cell, NAC is rapidly hydrolyzed to yield cysteine [18], which can regenerate total glutathione content and reduce excessively-oxidized glutathione levels. Another action of NAC is its direct scavenging ability against ROS [19]. Chronic administration of NAC increases ATP and GSH levels, and decreases lipid and protein oxidation in presynaptic terminals [20]. As currently available literatures indicate a strong relation between ROS and AD pathophysiology, it may be hypothesized that NAC would be an ideal antidote for AD. Therefore, the objective of the present work was to investigate and assess the possible neuroprotective effects of glutathione precursor, NAC, in a colchicine-induced AD-like disease in rats. Since AD involves the hippocampus, prefrontal cortices and the loss of cognitive functions, we investigated learning and memory and the expression of tau protein in the hippocampus and prefrontal cortices in AD model of rats treated with NAC.

## 2. Methods

### 2.1. Animals

In-house bred male albino *Wistar* rats, four months old and weighing 250–270 g, were used in this study. Rats were fed with water and food ad libitum. The rats were maintained under controlled conditions of light-dark cycle (12:12), temperature (22 ± 3 °C), humidity (50 ± 10%), and a pathogen free environment. A polypropylene cage with paddy husk as bedding material was used for housing the rats. The experimental procedure was approved by Institution Animal Ethics Committee (IAEC/KMC/2012).

### 2.2. Animal Groups

The rats were randomly divided into following five groups (*n* = 12 in each group): (i) Control-Rats in this group remained in the home cage without any surgical procedure, and treated with saline throughout the experimental period (2 weeks); (ii) Sham-Rats in this group underwent a sham surgical procedure, where skull surface was exposed, a bur hole was drilled aiming to the lateral ventricle, a 32 G needle was lowered into lateral ventricle, 5 µL of sterile artificial CSF was injected, and the needle was withdrawn, before the skin was sutured. These rats were treated with saline throughout the experimental period; (iii) Alzheimer’s like disease (AD)—rats in this group were injected with colchicine into the lateral ventricle stereotaxically (15 µg) to induce Alzheimer’s disease. These rats were treated with saline throughout the experiment; (iv) Rats with Alzheimer’s like disease treated with 50 mg/kg of NAC (AD + NAC-50)—rats in this group were injected with colchicine into the lateral ventricle stereotaxically to induce Alzheimer’s like disease, and were treated with NAC (50 mg/kg, i.p.) throughout the experiment, v) Rats with Alzheimer’s like disease treated with 100 mg/kg of NAC (AD + NAC-100)—rats in this group were injected with colchicine into the lateral ventricle stereotaxically to induce Alzheimer’s like disease, and were treated with NAC (100 mg/kg, i.p.) throughout the experiment.

### 2.3. Chemicals

NAC was purchased from Lobo chemicals (Mumbai, India). Artificial cerebrospinal fluid (ACSF: in *m* mol/L: 147 NaCl, 2.9 KCl, 1.6 MgCl_2_, 1.7 CaCl_2_ and 2.2 dextrose) was obtained from Biotech India Pvt. Ltd. (New Delhi, India). Colchicine was obtained from Sigma Aldrich (Sigma chemicals, St. Louis, MO, USA). Anti-tau-antibodies (ab32057) known to express in cytoplasm, cell membrane and axons of human, rats and mice neurons were obtained from abcam (Cambridge, MA, USA); all other chemicals and reagents were HPLC or analytical grade, and were obtained from from Sigma-Aldrich (Sigma chemicals, St. Louis, MO, USA).

### 2.4. Surgery and Intracerebroventricular Administration of Colchicine

To create an Alzheimer’s model, colchicine (a microtubule disrupting agent, also known to cause oxidative stress) was injected into the lateral ventricle (either left or right) stereotaxically. The stereotaxic surgical procedure was as described in our previous study [21]. Briefly, the rats were anesthetized with sodium pentobarbital (40 mg/kg, i.p.) and skull was exposed with a midline skin incision. A bur hole was drilled on the skull cap at the following stereotaxic coordinate: Anteroposterior—0.8 mm behind the bregma, Lateral—2 mm from midline [21]. The skull cap was drilled carefully up to the level of dura mater, without damaging any nervous tissue. A 32 G needle connected to one end of a capillary tube was held in the needle holder of the stereotaxic apparatus and inserted through the bur hole to a depth of 3.2 mm from skull surface aiming at the lateral ventricle. Other end of the capillary tube was connected to a Hamilton micro syringe filled with colchicine (or artificial cerebrospinal fluid for Sham group). Hamilton micro syringe was positioned in an infusion pump (Harvard apparatus). Five microliters of artificial cerebrospinal fluid or 15 µg colchicine in 5 µL of artificial cerebrospinal fluid was injected slowly over a period of 20 min. The needle was held in place for an additional 5 min before withdrawal, in order to prevent the backflow of the injected materials. Thereafter the needle was gently removed, and the scalp was closed with sutures. Antibiotics were applied on the surgical wound to prevent infection. The rats were kept in a warm place until they recovered from the anesthesia. Special care was taken during the post-operative period to provide food and water inside the cage of the rat. Following surgery, the rats were housed individually in cages until the end of the experiment.

### 2.5. NAC Administration

NAC in physiological saline was administered intraperitoneally, starting from one week prior to surgery and one week following the surgery at 50 mg/kg or 100 mg/kg dose. The doses of NAC were selected based on earlier studies [22], and human doses were calculated for rats. Twelve rats were used for cognitive tests in each group. Out of the twelve rats, six were randomly selected for Nissl staining, while the remaining six were for immunohistochemical studies.

### 2.6. Cognitive Assessment Using Passive Avoidance Task

To assess the cognitive functions in controls, AD, and AD + NAC treated rats, all rats were subjected to a passive avoidance test [23] at the end of treatment period. The apparatus consisted of a wooden box with a larger, bright compartment, and a smaller, dark compartment. The dark compartment was with a metal grid floor, which was connected to an electric shock stimulator. On the first day of test, rats were allowed to explore both bright and dark compartments of the apparatus for five minutes. This was followed by three test trials of five minutes each. In each trial, the fraction of time spent in each compartment was measured. In the fourth trial, as soon as the rat stepped into dark compartment, a foot shock (75 volts, 500 mA, DC current for 30 s) was given through the metal grid floor, and the rat was replaced to its home cage. Then, 24 h after foot shock, rats were placed in the bright compartment and the time taken to enter the dark compartment (Entry latency), and fraction of time spent bright and dark compartments, were measured with a digital timer. Normal rats avoid entering the dark chamber, where they received a shock on previous day, suppressing their normal behavior of exploring the dark compartment. Decreased/short latency to enter the dark compartment suggest poor memory retention.

### 2.7. Tissue Processing for Histopathological and Immuno Staining

A day after the passive avoidance test, rats in all groups were deeply anesthetized with ether and perfused with saline followed by freshly-prepared 4% paraformaldehyde in phosphate buffer (pH = 7.4). The brains were post fixed in 4% paraformaldehyde in phosphate buffer for 48 h. Brain tissues were processed for paraffin blocks. Coronal sections (7 µ) from prefrontal cortex and hippocampus region [24] were cut using a rotary microtome (Jung Biocutt 2035, Lieca, Wetztar, Germany). Five sections from each rat brain was mounted on gelatin coated slides and air dried. These paraffin sections were either stained with cresyl violet stain (Nissl stain) for histopathological observation and quantification of neurons in the prefrontal cortical regions and in the hippocampal sub regions or immunostained with anti-tau antibody for expression abnormal tau protein and neurofibrillary tangles in the prefrontal cortical regions and hippocampal sub regions. For cresyl violet staining, the sections were deparafinized in xylene for 5 min, and were rehydrated in descending grades of ethyl alcohol (100%, 90%, 70%, 50%) for 5 min each. Then, the sections were hydrated in distilled water for 5 min and stained with 0.1% aqueous cresyl violet stain at 60 °C for 20 min. Stained sections were differentiated in 70% ethyl alcohol to retain optimum staining. Finally, sections were dehydrated in two changes of 90% and absolute ethyl alcohol (5 min each). The sections were then air-dried for 10 min and cleared in two changes of xylene for 5 min each before being cover slipped with DPX.

### 2.8. Quantification of Neurons in Prefrontal Cortex and Hippocampus

The number of degenerating neurons in different regions of the prefrontal cortex and hippocampus were quantified in cresyl violet stained brain sections. Cresyl violet stains Nissl substance in the cytoplasm of neurons and it appears as granular purple-blue. For cells quantification, high quality images were captured with 40× objectives, with an Olympus digital camera (DP75) attached to an Olympus microscope. In each image, normal neurons were counted using NIS Elements Br version 4.30 software (Nicon Instruments Ins, New York, NY, USA) [21]. In each hippocampal photograph, a length of 350 µm of cornu ammonis sub-regions (CA1, CA2, CA3, and CA4) and 150 µ^2^ area of the dentate gyrus (DG) were selected for quantification. In the prefrontal cortex, the number of neurons in 250 µ^2^ area was counted in medial, lateral, and orbital regions of the prefrontal cortex. Neurons with a distinct profile and having a spherical nucleus were criteria for assessment as normal. Shrunken, deformed, indistinct borders between the nucleus and cytoplasm, pyknotic and hyperchromatic neurons were counted as degenerating neurons. Slides from different groups of rats were coded to avoid manual bias while counting the cells.

### 2.9. Immunostaining and Estimation of Expression of Tau and Neurofibrillary Tangles Formations

Neurofibrillary tangles were estimated according to the method described by Lu et al. [25]. Paraffin sections of the prefrontal cortex and hippocampus were immunostained for the expression of tau protein and neurofibrillary tangles. Sections were deparaffinized in xylene and rehydrated in descending grades of ethyl alcohol. Antigen retrieval was done by incubating the sections in 0.1 M citrate buffer at 60 °C for 30 min. The sections were treated with 3% H_2_O_2_ for 30 min to reduce the endogenous peroxidase activity in the tissue. Sections were then incubated for 30 min with 5% normal goat serum along with 0.3% Triton X-100 in PBS (pH = 7.4) to block the non-specific binding of the primary antibody. The sections were incubated with monoclonal rabbit Anti-tau antibody (E178) (1:1000, abcam—Cat. No. ab32057) overnight at 4 °C. The sections were incubated with biotinylated goat-anti-rabbit IgG as secondary antibody (1:200, Vector Laboratories, Burlingame, CA, USA) for 1 h at room temperature. The sections were washed in PBS and treated with avidin-biotin-peroxidase complex (ABC kit, Vector Laboratories, Burlingame, CA, USA) for 1 h at room temperature. Subsequently color was developed with 3,3′-diaminobenzidine as chromogen (DAB, Vector Laboratories, Burlingame, CA, USA) [25]. Throughout the staining, sections were washed three times with PBS after each incubation. To assess non-specific staining, several sections in each experiment were incubated in a buffer without primary antibodies. Sections were lightly counter stained with hematoxylin, dehydrated in ascending ethanol grades, cleared in xylene, and cover slipped in Permount (Fisher Scientific, Pittsburgh, PA, USA). High quality images were captured with 40× objective, with an Olympus digital camera (DP75) attached to an Olympus microscope (Olympus Scientific solutions, Waltham, MA, USA). In each image, immunostained neurons were counted using NIS Elements Br version 4.30 software. In each hippocampal section, 300 µm length of Cornu Ammonis sub regions (CA1, CA2, CA3, and CA4) and 300 µ^2^ area of the dentate gyrus (DG) were selected for quantification. In the prefrontal cortex, the number of neurons in 300 µ^2^ area were counted in medial, lateral and orbital regions of the prefrontal cortex. Slides from different groups of rats were coded to avoid manual bias while counting the cells.

### 2.10. Statistical Analysis

The data were expressed as Mean ± SD and analyzed with SPSS (version 25) statistical analysis software (IBM SPSS Statistics, Armonk, NY, USA). Data were analyzed with One-way ANOVA, followed by Bonferroni’s multiple comparison post hoc test. *p* values < 0.05 were considered as significant. The treatment effect between 2 doses of NAC were assessed through paired Student’s *t*-test.

## 3. Results

### 3.1. Passive Avoidance Test Performance

**Figure 1:**
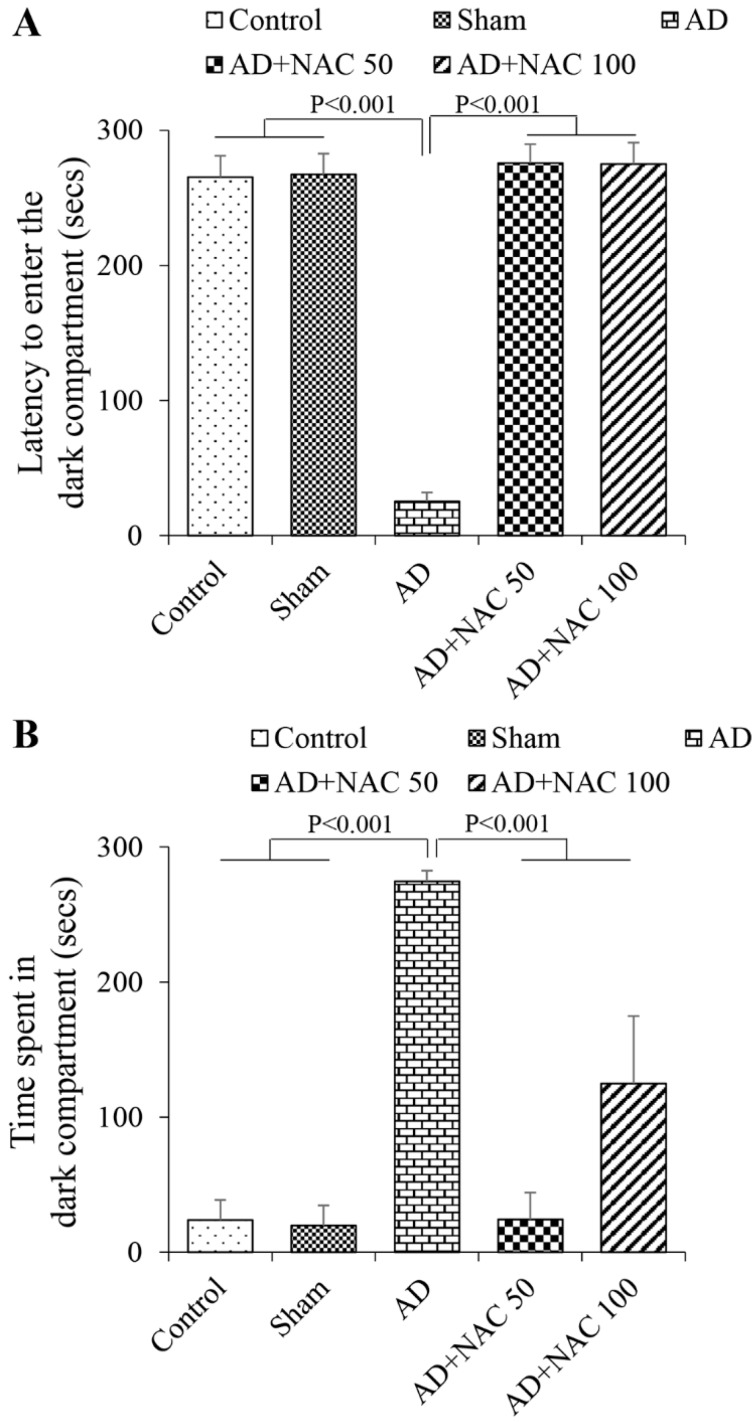
Performance of the rats in the passive avoidance task. (**A**) Latency to enter the dark compartment; (**B**) Time spent in the dark compartment during the retention test by the rats in different groups (Control, Sham operated, AD model (received intraventricular colchicine), AD + NAC 50 (AD model rats received 50 mg/kg of NAC, AD + NAC 100 (AD model rats received 100 mg/kg of NAC). Note that AD model rats had significantly shorter latencies to enter the dark compartment compared to control and sham group, and they were significantly higher in AD + NAC treated groups. Similarly, AD rats spent significantly more time in the dark compartment compared to control and sham groups, and time spent in dark compartment significantly decreased in the AD + NAC groups compared to the AD group (One-way ANOVA, Bonferroni’s multiple comparison test, *n* = 6 in all groups).

ICV colchicine resulted in a significant decrease in latency to enter the dark compartment (*p* < 0.001, Figure 1A) and a decrease in time spent in the dark compartment (*p* < 0.001, Figure 1B) in the passive avoidance task. However, sham operated group did not display any significant difference (*p* > 0.05) in these parameters. This demonstrates that ICV colchicine injections results in memory impairment. NAC treatment at both doses in the AD models of rats showed a significant increase in the latency to enter the dark compartment (*p* < 0.001, Figure 1A) and decrease in the time spent in the dark compartment (*p* < 0.001, Figure 1B). This demonstrates that NAC can reverse the memory impairment induced by ICV colchicine.

### 3.2. Neuronal Assay by Cresyl Violet Staining

**Figure 2:**
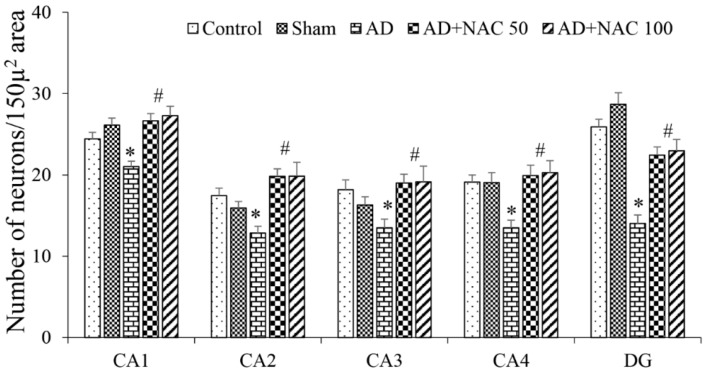
Quantitative estimation of normal neurons in various regions of the hippocampus. In the CA1, CA2, CA3, & CA4 regions, a length of 350 µm was selected; in the dentate gyrus (DG), 150 µ^2^ was selected for quantification. Note that in all regions, the number of neurons decreased significantly in AD group compared to control and Sham group, and was increased in AD + NAC treated groups compared to AD group. Values are expressed as mean ± SE. Control/sham vs. AD: *, *p* < 0.05–0.001; AD vs. AD + NAC 50 or AD vs. AD + NAC 100: #, *p* < 0.01 (One-way ANOVA, Bonferroni’s multiple comparison test, *n* = 6 in all groups).

**Figure 3:**
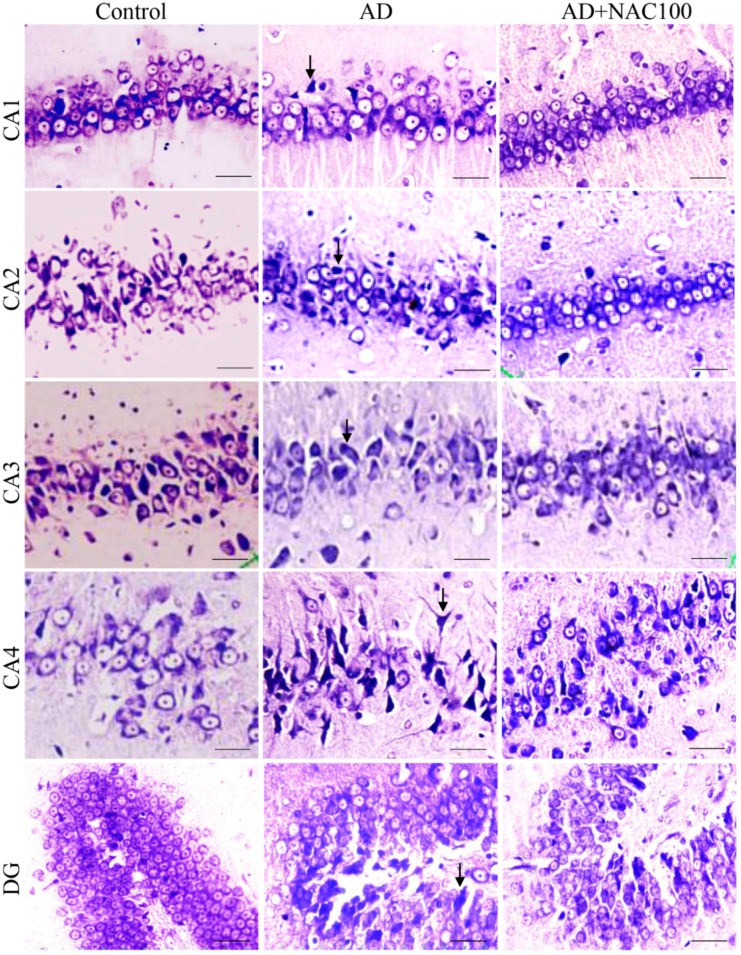
Photomicrographs of the hippocampal sub regions in different groups of rats stained with cresyl violet stain. Note the degenerating neurons (Arrow) in AD group in all the regions. No such degenerating neurons were observed in AD + NAC treated groups in any region. (Photomicrographs of Sham and AD + NAC 50 groups are avoided for simplicity). Scale bar = 20 µ, in CA1-CA4 regions, =15 µ in dentate gyrus (DG).

Hippocampus: There were no significant difference in quantitative and qualitative analysis between control versus sham operated group of rats. ICV colchicine significantly (*p* < 0.05) reduced number of normal neurons in all the regions of the hippocampus compared to control or sham operated rat groups (Figure 2). This neuronal loss was significantly greater in CA4 (*p* < 0.01) and DG (*p* < 0.001). The cells which were not considered for counting in cresyl violet staining were those neurons which showed many irregular cell membranes with signs of pyknotic and hyperchromatic. The border between the nucleus and cytoplasm was indistinct (Figure 3). These results indicate that ICV colchicine has a region-specific effect on the hippocampus. NAC at 50 or 100 mg/kg dose in colchicine administered rats showed significantly (*p* < 0.01) higher number of neurons in all the regions of hippocampus compared to rats treated with only colchicine (*p* < 0.01, AD models). This indicates that NAC has prevented colchicine-induced neuronal loss. There was no dose-dependent effect of NAC on the survival of neurons. There was no significant difference in number of neuronal expressions between control versus rats treated colchicine and NAC combination.

**Figure 4:**
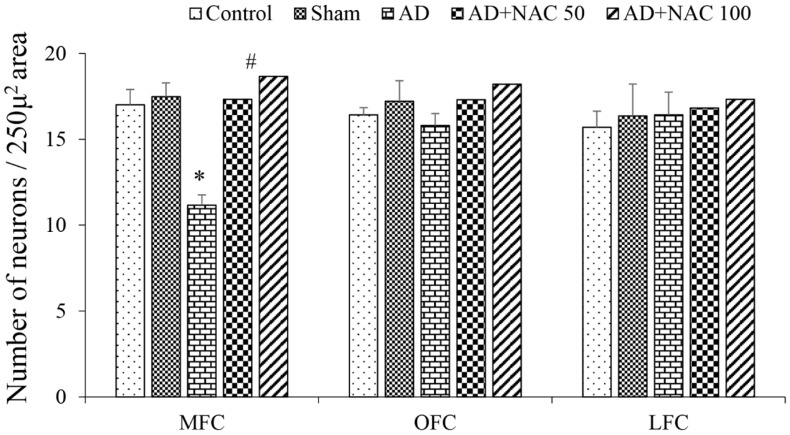
Quantitative estimation of normal neurons in prefrontal cortex. The number of neurons in 250 µ^2^ area was quantified. Values are expressed as mean ± SE. Control/sham vs. AD: *, *p* < 0.01; AD vs. AD + NAC 50 or AD vs. AD + NAC 100: #, *p* < 0.01 (One-way ANOVA, Bonferroni’s multiple comparison test, *n* = 6 in all groups). MFC—Media prefrontal cortex, OFC—Orbital prefrontal cortex, LFC—Lateral prefrontal cortex.

**Figure 5:**
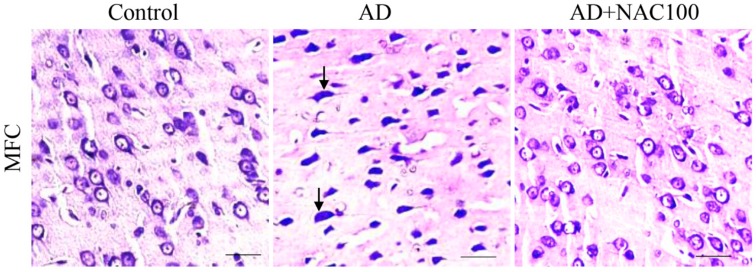
Photomicrographs of the prefrontal cortex in different groups of rats stained with Cresyl violet stain. Note the degenerating neurons (Arrow) in AD group. (Photomicrographs of Sham and AD + NAC 50 groups are avoided for simplicity). Scale bar = 20 µ. MFC—Media prefrontal cortex.

Prefrontal cortex: ICV colchicine significantly (*p* < 0.01) reduced the number of normal neurons in MFC compared to control rats or sham operated groups. However, such effects were not observed (*p* > 0.05) in lateral or orbito-prefrontal cortices (Figure 4). NAC at 50 or 100 mg/kg dose in colchicine administered rats showed significantly (*p* < 0.01) higher number of neurons NAC at 50 or 100 mg/kg dose showed an increase (*p* < 0.01) in survived neuronal numbers in MFC. These results demonstrate that ICV colchicine specifically affects MFC; no other regions of prefrontal cortex and NAC minimized this neuronal loss. The neurons of MFC showed signs of pyknotic and hyperchromasia (Figure 5).

### 3.3. Expression of Tau and Neurofibrillary Tangles

**Figure 6:**
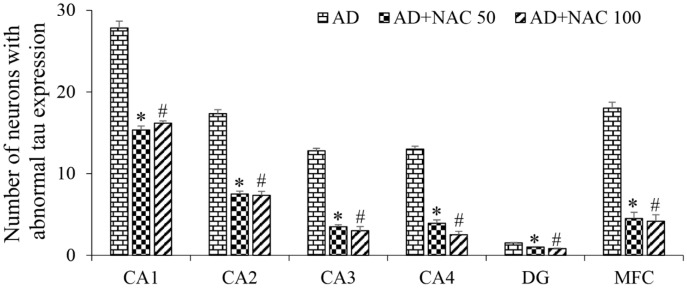
Quantitative estimation of neurofibrillary tangles (tau positive neurons) in the sub-regions of the hippocampus and prefrontal cortex. In CA1, CA2, CA3. & CA4 regions, a length of 300 µm was selected for quantification; in dentate gyrus (DG) and Media prefrontal cortex (MFC), a 300 µ^2^ area was selected. Note that in all regions, the number of tau positive neurons significantly decreased in AD + NAC treated groups compared to AD group. Values are expressed as mean ± SE. AD vs. AD + NAC 50: *, *p* < 0.001; AD vs. AD + NAC 100: #, *p* < 0.001 (One-way ANOVA, Bonferroni’s multiple comparison test, *n* = 6 in all groups).

**Figure 7:**
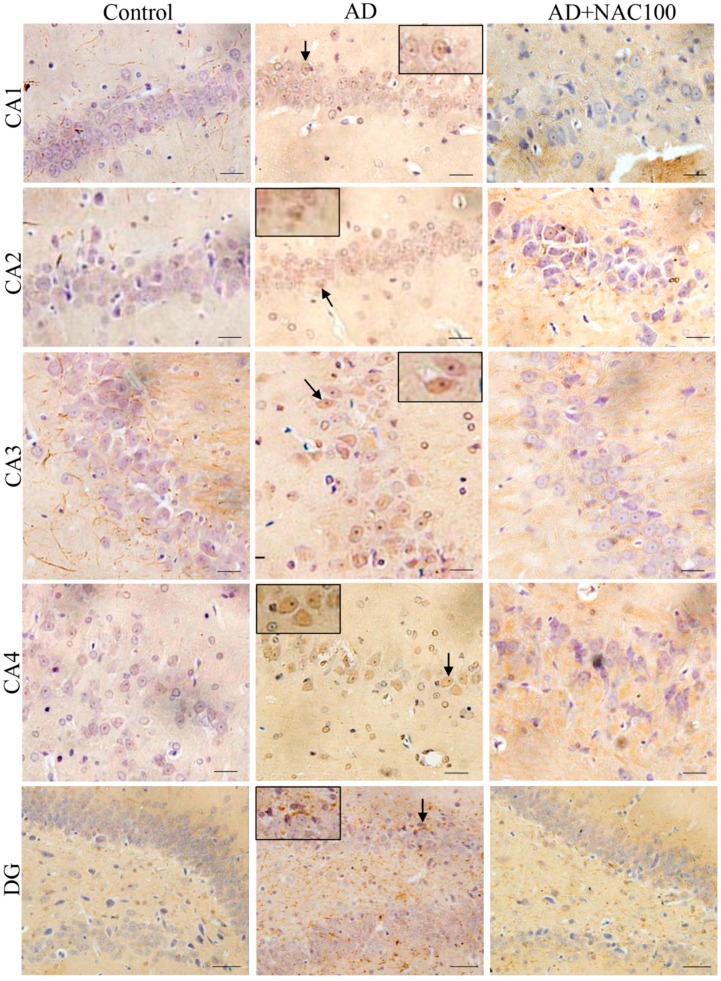
Photomicrographs of the hippocampal sub regions in different groups of rats immunostained for neurofibrillary tangles (anti-tau protein). Note the tau positive neurons (Arrow) in AD group. (Photomicrographs of Sham and AD + NAC 50 groups are avoided for simplicity). Scale bar = 40 µ, in CA1-CA4 regions, =35 µ in dentate gyrus (DG).

Hippocampus: Quantification of tau positive cells were significantly higher in all the regions of the hippocampus compared to the control or sham operated rats (*p* < 0.001, Figure 6). NAC at does of both 50 or 100 mg/kg has significantly reduced tau positive cells in all the regions of the hippocampus (*p* < 0.001, Figure 6). In control and sham operated rat brain sections, tau was hardly expressed. However, in rats treated with ICV colchicine, we observed an accumulation of tau-positive foci in CA1, CA2, and CA3 regions of the hippocampus, and to a lesser extent, in the CA4 and dentate gyrus regions. Upon closer examination, this atypical tau immunostaining design was localized to the cell bodies having large nuclei with limited axonal projections (Figure 7). Taken together, these results indicate that ICV colchicine induces higher tau expression, and that NAC has reduced their accumulation in neurons.

**Figure 8:**
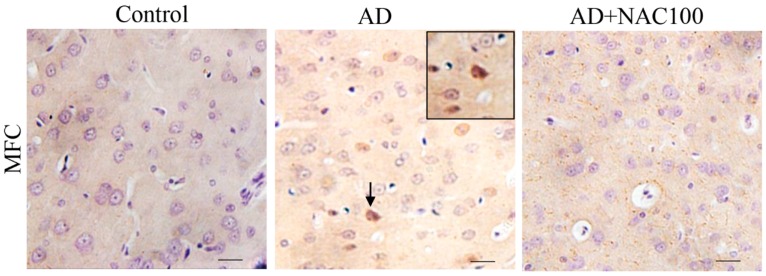
Photomicrographs of the prefrontal cortex in different groups of rats immunostained for neurofibrillary tangles (anti-tau protein). Note the tau positive neurons (Arrow) in AD group. (Photomicrographs of Sham and AD + NAC 50 groups are avoided for simplicity). Scale bar = 40 µ. MFC—Media prefrontal cortex.

Prefrontal cortex: The number of tau positive cells were significantly higher in medial prefrontal cortex compared to control or sham operated group (*p* < 0.001, Figure 6). NAC treatment in AD rats significantly reduced these tau positive cells (*p* < 0.001, Figure 6). These results demonstrate that NAC can reduce or minimize the abnormal tau expression in the medial prefrontal cortex. Qualitative analysis of rat brain sections revealed that medial prefrontal cortex has highly expressed tau positive cells, but not lateral or orbito-frontal cortices (Figure 8).

## 4. Discussion

This study investigated the effect of NAC in the prevention of memory loss observed in AD using an established animal model of AD. The major findings of this study are that pre and post treatment of NAC improved cognition, and reduced neuronal loss and tau expression in specific regions of the brain.

ICV colchicine administration is known to cause disproportionate free radical generation and oxidative damage [26]. Hence, cognitive loss in the form of memory impairment observed in the present study can be positively correlated. In the present study, NAC treatment was able to improve the cognitive deficit in the AD model of rats. It’s well known that oxidative stress is the major causative factor for creating neuronal-change-mediated behavioral deficits in AD [27].

ICV colchicine causes oxidative stress by enhancing GLU/GABA ratio and NOS production in the brain. This causes extreme glutamate activity and NO production, thereby causing neuronal loss [28]. In addition to this, colchicine binds to tubulin protein of the microtubule, causing its depolymerization and weakening with consequent block of axonal transport and mitosis, resulting in neuronal loss. In the present study, ICV colchicine resulted in neuronal loss in all the regions of the hippocampus and MFC. This can be correlated with both oxidative stress and tubulin protein dysfunctions. Central colchicine inducing elevated biomarkers of oxidative stress is well reported [29]. Hence, we investigated tubulin protein dysfunction by evaluating tau protein expression. Normally, tau protein exists in a balance of phosphorylation and dephosphorylation in the human brain. Abnormal tau hyperphosphorylation impairs its binding to microtubules, and prevents microtubule assembly in the neurons. This results in NFT and impaired transport along the axonal microtubule [11]. Excessive or abnormal phosphorylation of tau results in the transformation of normal tau into phosphorylated tau and NFT. Oxidative stress has been shown to also promote tau hyperphosphorylation [30].

Oxidative stress is also attributed to tau abnormalities. The manipulation of antioxidant defenses affects the longevity of neurons in several animal models including AD [31]. Oxidative stress is now seen as a further hallmark of tau pathology in both patients and animal models. The ICV colchicine-induced AD model with oxidative stress is well established, but whether this oxidative stress results in tau pathology is not known. In the present study, ICV colchicine induced NFT in all the regions the hippocampus and MFC. We further correlate that increased tau expression in neuronal cytoplasm in the hippocampus and MFC was associated with cognitive dysfunction. This could be due to a change in the tau phosphorylation in hippocampal neurons. These phosphorylated taus in the neurons inhibit axoplasmic transportation, neural transmission, and synaptic activity which eventually result in cognitive impairment. Excessive phosphorylated tau protein accumulates in the cell body of degenerated neurons, and is positively correlated with the degree of clinical dementia in patients with AD [32].

In AD, abnormal hyperphosphorylated tau pathology is known to begin from the entorhinal cortex, proceed to the hippocampus and frontal and temporal cortices, and lastly to all isocortex areas. The mechanism of differential vulnerability of specific regions of the brain to tau pathology is not clearly understood. It could be due to region-specific expression of tau pathology related proteins like tau phosphatases and kinases. Further hippocampal tau pathology is related to neuroanatomical connections [33,34]. Lace and co-workers demonstrated tau pathology specifically in hippocampal input regions and projection zones such as entorhinal cortex, CA1 region, and the molecular layer of the dentate gyrus. The junction between CA1 and the subiculum showed dense tau expression. Tau-induced pathology is thought to then spread progressively to the hippocampal formation. According to this, the main target region is perforant pathway, i.e., the DG, CA3, CA1, and subiculum [35]. Another study showed a conspicuous abnormal phosphorylation of tau in the typical thorny excrescences of hippocampal CA3 neurons [36]. Since thorny outgrowths represent a major synaptic target of granule cell axons (mossy fibers), we suggested that such abnormal phosphorylation may play a role in the memory impairment which is typical of AD patients.

AD is known to involve cholinergic basal forebrain cortical projection [37] with cholinergic neuronal loss [38], which is characterized by deficits in memory and cognition. Colchicine causes a loss of cholinergic neurons, damage of cholinergic pathways, and the reduction in cholinergic turnover [6]. Loss of cholinergic pathways correlates with cognitive dysfunction in AD [39]. It has been shown that blocking CA3 cholinergic receptors impairs the encoding of information, resulting in memory loss [40]. The prefrontal cortex performs higher executive functions including working memory [41] and retrieval from long term memory [42]. In rodents, the MFC has been shown to be significant part in working memory [43] and attention [44]. This part of the pre frontal cortex roughly corresponds to the dorsolateral prefrontal cortex in humans and other primates [45]. Lesions of this region result in severe deficits in attention [46] and memory loss. Medial and orbitofrontal cortices [13] receive direct input from the CA1 region of the hippocampus [14]. Because of these connections, the frontal cortex, especially the medial and orbital, becomes pathologically involved in dementia associated with AD [15]. According to the connection hypothesis postulated by many workers, atrophy might also progress to the MFC. In our study, neuronal loss, as well as tau positive neurons, were observed in all the regions of the hippocampus and also medial prefrontal cortex.

Relative to its high oxygen ingestion, the antioxidant capacity of the brain is lowered. ROSs, when they accumulate, can cause neuronal degeneration due to the large volume of unsaturated fatty acids in neuronal cell membranes [47]. Adequate studies clearly demonstrated the fact that oxidative stress in any form can harm the neurons. Hence, the application of antioxidants such as NAC would play a vital role in maintaining neuronal homeostasis [48]. In the present study, NAC treatment has minimized memory loss with reduction in tau immunoreactivity and neuronal loss in hippocampus and MFC. NAC is known to exert its neuroprotective potential through two well-known mechanisms, that is, restoration of glutathione pool [49], and a direct scavenging ability against ROS [19]. After entering the neuron, it rapidly hydrolyzed to yield cysteine, which can regenerate total glutathione contents and reduce excessively-oxidized glutathione. In addition to this, NAC is known to increase ATP and glutathione levels, and decrease lipid and protein oxidation [20]. Preclinical data also provide evidence that NAC treatment is beneficial in AD murine models counteracting oxidative damage [50]. Further, Tucker and co-workers [51] demonstrated anti-amyloid efficacy by NAC. However, there is not much research which has demonstrated the anti-tau efficacy of NAC in an animal model study except for few studies. Du and co-workers [52] demonstrated the ameliorative effects of NAC on the hippocampal accumulation of tau., where they found a reduction in tau immunoreactivity in mossy cells and oligodendrocytes after NAC treatment. It is well known that over activation of CDK5 and GSK3B kinases enhances the formation of neurofibrillary tangles in AD brain by hyperphosphorylation of tau protein. An interesting study by Farr and co-workers [53], where GSK3B level was suppressed in SAMP8 mice, was associated with an improvement in learning and memory decreased oxidative stress. Hence, boosting antioxidant defense by NAC could inhibit GSK3B. Our study clearly demonstrates that NAC can minimize the expression of intra-neuronal tau and also minimize tau-related memory loss after central colchicine administration in rats. Further investigating the role of NAC on caspase activity in minimizing tau aggregation would be an ideal approach in future. There is reason to believe that NAC could inhibit caspase activity during the progression of AD, where tau tend to aggregate. Tau catalyzes auto-acetylation mediated by a pair of catalytic sensitive cysteine residues in its microtubule-binding domain [54]. The tau aggregation inhibitor, methyl thioninium, inhibited the formation of tau filaments and their toxic precursors through oxidation of cysteine residues by preventing the formation of disulfide bridges [55]. The mucolytic activity of NAC is due to its ability to break disulphide bridge. Chemical features explaining the efficient disulphide breaking activity of NAC were also explained [56]. Hence, exploring the ability of NAC in these contexts would be the ideal approach for the future.

## 5. Conclusions

Tau expression in the form of NFT in neuronal cell bodies in AD is due to microtubule dysfunction which affects neuronal signaling. This study demonstrates that NAC prevents tau abnormality in the neurons, thereby minimizing neuronal loss and cognitive deficits. Hence, measures to build antioxidant defense, especially glutathione in brain with early AD signs would be a possible approach.

## References

[1] Huang W.J., Zhang X., Chen W.W. (2016). Role of oxidative stress in Alzheimer’s disease. Biomed. Rep..

[2] Torres L.L., Quaglio N.B., de Souza G.T., Garcia R.T., Dati L.M.M., Moreira W.L., de Melo Loureiro A.P., de souza-Talarico J.N., Smid J., Porto C.S. (2011). Peripheral oxidative stress biomarkers in mild cognitive impairment and Alzheimer’s disease. J. Alzheimer’s Dis..

[3] Shigematsu K., McGeer P.L. (1992). Accumulation of amyloid precursor protein in damaged neuronal processes and microglia following intracerebral administration of aluminum salts. Brain Res..

[4] Kumar M.H.V., Gupta Y.K. (2002). Intracerebroventricular administration of colchicine produces cognitive impairment associated with oxidative stress in rats. Pharmacol. Biochem. Behav..

[5] Zilka N., Novak A. (2006). The tangled story of Alois Alzheimer. Bratisal. Lek. List..

[6] Meyers C.A., Kudelka A.P., Conrad C.A., Gelke C.K., Grove W., Pazdur R. (1992). Neurotoxicity of CI-980, a novel mitotic inhibitor. Clin. Cancer Res..

[7] Ganguly R., Guha D. (2008). Alteration of brain monoamines & EEG wave pattern in rat model of Alzheimer’s disease & protection by *Moringa oleifera*. Indian J. Med. Res..

[8] Nakayama T., Sawada T. (2002). Involvement of microtubule integrity in memory impairment caused by colchicine. Pharmacol. Biochem. Behav..

[9] Dahl D.A., Bignami A., Bich N.T., Chi N.H. (1980). Immunohistochemical characterization of neurofibrillary tangles induced by mitotic spindle inhibitors. Acta Neuropathol..

[10] Merrick S.E., Demoise D.C., Lee V.M. (1996). Site-specific dephosphorylation of tau protein at Ser202/Thr205 in response to microtubule depolymerization in cultured human neurons involves protein phosphatase 2A. J. Biol. Chem..

[11] Alavi Naini S.M., Soussi-Yanicostas N. (2015). Tau Hyperphosphorylation and Oxidative Stress, a Critical Vicious Circle in Neurodegenerative Tauopathies?. Oxid. Med. Cell. Longev..

[12] Kang S.W., Kim S.J., Kim M.S. (2017). Oxidative stress with tau hyperphosphorylation in memory impaired 1,2-diacetylbenzene-treated mice. Toxicol. Lett..

[13] Kahn I., Andrews-Hanna J.R., Vincent J.L., Snyder A.Z., Buckner R.L. (2008). Distinct cortical anatomy linked to subregions of the medial temporal lobe revealed by intrinsic functional connectivity. J. Neurophysiol..

[14] Laroche S., Davis S., Jay T.M. (2000). Plasticity at hippocampal to prefrontal cortex synapses: Dual roles in working memory and consolidation. Hippocampus.

[15] Thompson P.M., Hayashi K.M., Dutton R.A., CHIANG M.C., Leow A.D., Sowell E.R., de Zubicaray G., Becker J.T., Lopez O.L., Aizenstein H.J. (2007). Tracking Alzheimer’s disease. Ann. N. Y. Acad. Sci..

[16] Cente M., Filipcik P., Mandakova S., Zilka N., Krajciova G., Novak M. (2009). Expression of a truncated human tau protein induces aqueous-phase free radicals in a rat model of tauopathy: Implications for targeted antioxidative therapy. J. Alzheimer’s Dis..

[17] Park S.Y., Kim H.S., Cho E.K., Kwon B.Y., Phark S., Hwang K.W., Sul D. (2008). Curcumin protected PC12 cells against beta-amyloid-induced toxicity through the inhibition of oxidative damage and tau hyperphosphorylation. Food Chem. Toxicol..

[18] Sen O., Caner H., Aydin M.V., Ozen O., Atalay B., Altinors N., Bavbek M. (2006). The effect of mexiletine on the level of lipid peroxidation and apoptosis of endothelium following experimental subarachnoid hemorrhage. Neurol. Res..

[19] Kerksick C., Willoughby D. (2005). The Antioxidant role of glutathione and N-acetyl-cysteine supplements and exercise-induced oxidative stress. J. Int. Soc. Sports Nutr..

[20] Banaclocha M.M. (2001). Therapeutic potential of N-acetyl-cysteine in age-related mitochondrial neurodegenerative diseases. Med. Hypothesis.

[21] Madhyastha S., Somayaji S.N., Rao M.S., Nalini K., Bairy K.L. (2002). Hippocampal brain amines in methotrexate-induced learning and memory deficit. Can. J. Physiol. Pharmacol..

[22] Farr S.A., Poon H.F., Dogrukaol-Ak D., Drake J., Banks W.A., Eyerman E., Butterfield D.A., Morley J.E. (2003). The antioxidants α-lipoic acid and *N*-acetylcysteine reverse memory impairment and brain oxidative stree in aged SAMP8 mice. J. Neurochem..

[23] Bures J., Burešová O., Huston J.P. (1983). Techniques and Basic Experiments for the Study of Brain and Behavior.

[24] Pelligrino L.J., Pelligrino A.S., Cushman A.J. (1981). Stereotaxic Atlas of the Rat Brain.

[25] Lu F., Li X., Suo A.Q., Zhang J.W. (2010). Inhibition of tau hyperphosphorylation and beta amyloid production in rat brain by oral administration of atorvastatin. Chin. Med. J..

[26] Kumar A., Dogra S., Prakash A. (2009). Neuroprotective Effects of *Centella asiatica* against Intracerebroventricular Colchicine-Induced Cognitive Impairment and Oxidative Stress. Int. J. Alzheimer’s Dis..

[27] Cantuti-Castelvetri I., Shukitt-Hale B., Joseph J.A. (2000). Neurobehavioral aspects of antioxidants in aging. Int. J. Dev. Neurosci..

[28] Kumar A., Naidu P.S., Seghal N., Padi S.S.V. (2007). Effect of curcumin on intracerebroventricular colchicine-induced cognitive impairment and oxidative stress in rats. J. Med. Food.

[29] Mohammed A.R., Soliman G.Y., Ismail C.A., Mannaa H.F. (2015). Neuroprotective role of vitamin D3 in colchicine-induced Alzheimer’s disease in rats. Alexandria J. Med..

[30] Costa A.P., Tramontina A.C., Biasibetti R., Batassini C., Lopes M.W., Wartchow K.M., Bernardi C., Tortorelli L.S., Leal R.B., Gonçalves C.-A. (2012). Neuroglial alterations in rats submitted to the okadaic acid-induced model of dementia. Behav. Brain Res..

[31] Borza L.R. (2014). A review on the cause-effect relationship between oxidative stress and toxic proteins in the pathogenesis of neurodegenerative diseases. Med. Surg. J..

[32] Iqbal K., Liu F., Gong C.X., Grundke-Iqbal I. (2010). Tau in Alzheimer disease and related tauopathies. Curr. Alzheimer Res..

[33] Lace G., Savva G.M., Forster G., De Silva R., Brayne C., Matthews F.E., Barclay J.J., Dakin L., Ince P.G., Wharton S.B. (2009). Hippocampal tau pathology is related to neuroanatomical connections: An ageing population-based study. Brain.

[34] Jucker M., Walker L.C. (2013). Self-propagation of pathogenic protein aggregates in neurodegenerative diseases. Nature.

[35] Llorens-Martin M., Blazquez-Llorca L., Benavides-Piccione R., Rabano A., Hernandez F., Avila J., DeFelipe J. (2014). Selective alterations of neurons and circuits related to early memory loss in Alzheimer’s disease. Front. Neuroanat..

[36] Blazquez-Llorca L., Garcia-Marin V., Merino-Serrais P., Ávila J., DeFelipe J. (2011). Abnormal tau phosphorylation in the thorny excrescences of CA3 hippocampal neurons in patients with Alzheimer’s disease. J. Alzheimer’s Dis..

[37] Mufson E.J., Ginsberg S.D., Ikonomovic M.D., DeKosky S.T. (2003). Human cholinergic basal forebrain: Chemoanatomy and neurologic dysfunction. J. Chem. Neuroanat..

[38] Wevers A., Witter B., Moser N., Burghaus L., Banerjee C., Steinlein O.K., Schütz U., de Vos R.A.I., Steur E.N.H.J., Lindstrom J. (2000). Classical Alzheimer features and cholinergic dysfunction: Towards a unifying hypothesis?. Acta Neurol. Scand..

[39] Kim H.J., Moon W.J., Han S.H. (2013). Differential cholinergic pathway involvement in Alzheimer’s disease and subcortical ischemic vascular dementia. J. Alzheimer’s Dis..

[40] Rogers J.L., Kesner R.P. (2004). Cholinergic modulation of the hippocampus during encoding and retrieval of tone/shock-induced fear conditioning. Learn. Mem..

[41] Funahashi S. (2013). Thalamic mediodorsal nucleus and its participation in spatial working memory processes: Comparison with the prefrontal cortex. Front. Syst. Neurosci..

[42] Tomita H., Ohbayashi M., Nakahara K., Hasegawa I., Miyashita Y. (1999). Top-down signal from prefrontal cortex in executive control of memory retrieval. Nature.

[43] Rossi M.A., Hayrapetyan V.Y., Maimon B., Mak K., Je H.S., Yin H.H. (2012). Prefrontal cortical mechanisms underlying delayed alternation in mice. J. Neurophysiol..

[44] Euston D.R., Gruber A.J., Mcnaughton B.L. (2012). The role of medial prefrontal cortex in memory and decision making. Neuron.

[45] Farovik A., Dupont L.M., Arce M., Eichenbaum H. (2008). Medial prefrontal cortex supports recollection, but not familiarity, in the rat. J. Neurosci..

[46] Kahn J.B., Ward R.D., Kahn L.W., Rudy N.M., Kandel E.R., Balsam P.D., Simpson E.H. (2012). Medial prefrontal lesions in mice impair sustained attention but spare maintenance of information in working memory. Learn. Mem..

[47] Clark T.A., Lee H.P., Rolston R.K., Zhu X., Marlatt M.W., Castellani R.J., Nunomura A., Casadesus G., Smith M.A., Lee H. (2010). Oxidative stress and its implications for future treatments and management of Alzheimer disease. Int. J. Biomed. Sci..

[48] Underwood B.R., Imarisio S., Fleming A., Rose C., Krishna G., Heard P., Quick M., Korolchuk V.I., Renna M., Sarkar S. (2010). Antioxidants can inhibit basal autophagy and enhance neurodegeneration in models of polyglutamine disease. Hum. Mol. Genet..

[49] Aoyama K., Suh S.W., Hamby A.M., Liu J., Chan W.Y., Chen Y., Swanson R.A. (2006). Neuronal glutathione deficiency and age-dependent neurodegeneration in the EAAC1 deficient mouse. Nat. Neurosci..

[50] Tchantchou F., Graves M., Rogers E., Ortiz D., Shea T.B. (2005). *N*-acteylcysteine alleviates oxidative damage to central nervous system of ApoE-deficient mice following folate and vitamin E-deficiency. J. Alzheimer’s Dis..

[51] Tucker S., Ahl M., Bush A., Westaway D., Huang X., Rogers J.T. (2005). Pilot study of the reducing effect on amyloidosis in vivo by three FDA pre-approved drugs via the Alzheimer’s APP 5′ untranslated region. Curr. Alzheimer Res..

[52] Du X., West M.B., Cheng W., Ewert D.L., Li W., Saunders D., Towner R.A., Floyd R.A., Kopke R.D. (2016). Ameliorative Effects of Antioxidants on the Hippocampal Accumulation of Pathologic Tau in a Rat Model of Blast-Induced Traumatic Brain Injury. Oxid. Med. Cell. Longev..

[53] Farr S.A., Ripley J.L., Sultana R., Zhang Z., Niehoff M.L., Platt T.L., Murphy M.P., Morley J.E., Kumar V., Butterfield D.A. (2016). Antisense oligonucleotide against GSK-3B in brain of SAMP8 mice improves learning and memory and decreases oxidative stress: Involvement of transcription factor Nrf2 and implication for Alzheimer disease. Free Radic. Biol. Med..

[54] Cohen T.J., Friedmann D., Hwang A.W., Marmorstein R., Lee V.M. (2013). The microtubuleassociated tau protein has intrinsic acetyltransferase activity. Nat. Struct. Mol. Biol..

[55] Wegmann S., Medalsy I.D., Mandelkow E., Müller D.J. (2012). The fuzzy coat of pathological human Tau fibrils is a two-layered polyelectric brush. Proc. Natl. Acad. Sci. USA.

[56] Aldini G., Altomare A., Baron G., Vistoli G., Carini M., Borsani L., Sergio F. (2018). N-Acetylcysteine as an antioxidant and disulphide breaking agent: The reasons why. Free Radic. Res..

